# Effect of antenatal care and social well-being on early neonatal mortality in Bangladesh

**DOI:** 10.1186/s12884-018-2129-y

**Published:** 2018-12-10

**Authors:** Sanjit Roy, Md. Aminul Haque

**Affiliations:** Department of Population Sciences, University of Dhaka, Dhaka, 1000 Bangladesh

**Keywords:** Early neonatal mortality, Antenatal care, Social well-being, Effect, Bangladesh

## Abstract

**Background:**

Bangladesh has achieved MDG 4, but although post neonatal and child mortality have shown impressive declines, neonatal mortality is still lagging behind. More efforts have to be made to improve this state of affairs. The objective of this paper is to identify the effect of proper antenatal care and social wellbeing on early neonatal mortality in Bangladesh.

**Methods:**

The data used for this study is the Bangladesh Multiple Indicator Cluster Survey. This study used several independent variables such as mother’s school attendance, receiving antenatal care, receiving TT injection, place of residence and wealth quintile. Here both bivariate and multivariate analysis have been used. At bivariate level, simple cross tabulation and appropriate measures of association have been used to find out the statistical association between dependent and independent variables. In this study the outcome/dependent variable is early neonatal mortality (children who died within 7 days after birth) which is a binary variable. If early neonatal mortality occurs among the respondents then it is considered as 1, otherwise it is considered as 0. Logistic regression was used to identify the factors which are involved in reducing this early neonatal mortality.

**Results:**

Women who received antenatal care during their time of pregnancy are likely to have 18% lower odds of experiencing early neonatal mortality (OR = 0.82, CI = (0.71–0.95)) compared to groups who did not receive antenatal care during pregnancy. In terms of social well-being, the woman who comes from the richest family are likely to have 45% lower odds of experiencing early neonatal mortality (OR = 0.55, CI = (0.42–0.720) compared to the poorest one.

**Conclusion:**

The outcomes of this paper suggest that the women’s antenatal care and social well-being has a significant effect on early neonatal mortality.

## Background

Reduction of child mortality was one of the major goals of MDGs and its target was to reduce childhood mortality rate up to 66% within the year 2015 [1]. With the target of sustainable development goals (SGD) for achieving universal health coverage, neonatal mortality must decline in tandem with child mortality. Childhood mortality is a very prominent factor in the public health sector of any country (Mustafa and Odimegwu 2008). As a cause of childhood mortality, including early neonatal mortality, different socio-economic factors, socio-demographic factors, nutritional factors of the mothers are involved [2] and other factors may also be responsible [3].

The infant mortality rate was 94/1000 live births and the under-five child mortality was 151/1000 live births in the year 1990 [4]. But just after 6 years both infant mortality rate and under five child mortality rate have declined with IMR at 46/1000 live births and under-5 mortality rate at 58/1000 live births in the year 2015 [5]. According to the Health Bulletin 2017 of DGHS, the neonatal mortality rate is reported as 19 per 1000 live births (SVRS 2016) and 28 per 1000 live births (BDHS 2014) [6]. The neonatal period (first 28 days of life) is the most vulnerable time for a child. But recently the neonatal mortality is decreasing worldwide as well as in Bangladesh, where the neonatal mortality rate has decreased from 36 to 19 deaths per 1000 live births [7]. However, the decreasing rate of neonatal mortality is slower compared to post-neonatal mortality rate. So, more emphasis has to give to improve this state of affairs.

The most significant indicator of child well-being is the under-five child mortality rate including good health and nutritional status of the children across the world [8].

The rate of child mortality is decreasing globally. For example, during the year 1990, the rate of child mortality has declined across all the regions under WHO with the exception of Western Pacific Regions [8]. Despite this decreasing pattern, child survival remains an urgent concern. In spite of the subsequent development of health sectors, still a significant percentage of children (i.e. 17,000/per day) die before reaching their fifth birthday and this rate of childhood mortality is significantly higher in the low income countries compared to high income countries [9]. According to UNICEF, in 2013 the rate of under-five child mortality for low income countries was 76/1000 live births and this rate was approximately twelve times higher than high income countries [10].

Overall it has been found that across the globe the neonatal mortality is decreasing but this rate of decline is slower compared to post neonatal mortality. In the total life span for a human being the first 28 days of life are the most vulnerable time period for his survival. For that reason, more efforts should be made to reduce early neonatal mortality. Bangladesh is not an exception to this trend of childhood mortality. So, by considering all these issues, this paper aims to find out the effect of antenatal care and social well-being on early neonatal mortality in the context of Bangladesh.

## Methods

### Source of data

In this study the Bangladesh Multiple Indicator Cluster Survey [5] data was used which was conducted from December 2012 to March 2013 with the collaboration of the Bangladesh Bureau of Statistics (BBS) and Ministry of Planning. The survey used the multistage stratified sampling method for collecting data. Both technical and financial support for conducting this survey was provided by UNICEF, Bangladesh. The number of respondents who were both ever married and ever given birth was 44,207 and belonged to the reproductive age group.

### Model specification

Here, early neonatal mortality has been defined as neonates who died before reaching age 7 days after birth. In this study, early neonatal mortality has been considered as the dependent variable. On the other hand the independent variables are: Maternal related factors: School attendance or literacy, Received antenatal care, Received TT injection, Socioeconomic factors: Place of residence and Wealth index.

The proposed hypothesis of this study includes:

- Hypothesis 1: The mothers who are receiving sufficient antenatal care have lower probability of early neonatal mortality (ENM).
- Hypothesis 2: The mothers with higher socio-economic status have lower probability of ENM.

### Statistical methods

In this study both bivariate and multivariate analyses have been used. At bivariate level, simple cross tabulation and appropriate measures of association have been applied i.e. chi-square test have been used to find out the statistical association (which are considered statistically significant at 5% level) between dependent and independent variables. Here, the outcome/dependent variable is early neonatal mortality (ENM) and it is considered as a dichotomous variable indicating 1 (if occurred) and 0 (if not occurred). Logistic regression was used to identify the factors which involved reducing this early neonatal mortality. The entire analyses have been conducted using the statistical software STATA.

## Results

**Fig. 1:**
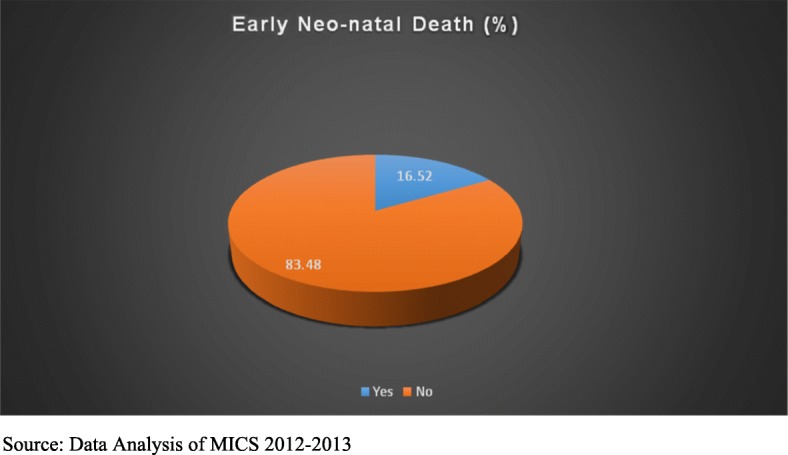
Early Neo-natal Death (%)

In this study, around 17.0% respondents reported that they ever had experience of early neonatal mortality in their reproductive life-span (Fig. 1).

The average age of the study population is 29 years. Women’s school attendance is an important factor to reduce early neonatal mortality. In this study around 72.2% respondents reported that they had ever attended school and the remaining 27.8% stated that they never attended school. In terms of place of residence, only 17.3% respondents came from urban areas and 82.7% from the rural areas. In terms of wealth index of the respondents, 22.7% are poorest, 20.8% are poorer, 20.1% are less poor, 19.6% are richer and the remaining 16.7% comes from the richest family. Among the respondents around 65.2% reported that they received antenatal care during their time of pregnancy and the remaining 34.9% did not. Approximately 59.1% respondents reported that they received TT injection during their pregnancy, whereas 40.9% did not (Table 1).

**Table 1:** Socio-demographic characteristics of the respondents

Background characteristics	Number of respondents (%)
Average Age of the Respondent	Mean (SD) 29.72 (9.46)
Ever Attended School	
Yes	37,315 (72.22)
No	14,357 (27.78)
Total	51,627 (100.00)
Received Antenatal Care	
Yes	5051 (65.15)
No	2702 (34.85)
Total	7753 (100.00)
Received TT Injection	
Yes	4581 (59.09)
No	3172 (40.91)
Total	7753 (100.00)
Place of Residence	
Urban	8925 (17.27)
Rural	42,747 (82.73)
Total	51,672 (100.00)
Wealth Index	
Poorest	11,744 (22.73)
Second	10,769 (20.84)
Middle	10,392 (20.11)
Fourth	10,118 (19.58)
Richest	8649 (16.74))
Total	51,627 (100.00)

The early neonatal mortality is influenced by many factors. According to the findings of this study women’s school attendance shows a significant association (*p* < 0.001) with early neonatal mortality. This study found that the women who ever attended school has lower percentage of early neonatal mortality (11.8%) compared to women who never attended school (26.8%).Women who received antenatal care during their pregnancy has significantly (*p* < 0.001) lower percentage of early neonatal mortality (ENM) (10.2%) compared to women who never received antenatal care during their pregnancy (15.6%). The study also found significant association (*p* < 0.001) between receiving TT injection and early neonatal mortality. According to the findings of this study around 10.7% women reported early neonatal mortality although they had received TT injection, in comparison to 14.0% women who had never received TT injection.

In terms of place of residence, women who live in rural areas have significantly higher (*p* < 0.001) early neonatal mortality (17.2%) compared to the women living in urban areas (13.0%).Wealth index has also statistically significant impact (*p* < 0.001) on early neonatal mortality (ENM). This study found that ENM is higher in the poorest groups (20.8%) compared to the richest groups (10.1%) (Table 2).

**Table 2:** Percentage of Childhood mortality/Early stage neo-natal mortality by different factors

Childhood mortality/ early stage neo-natal mortality			
Factors	Yes	No	*P*-value
	N (%)	N (%)	
Ever Attended School (*N* = 44,207)			
Yes	3554 (11.76)	26,667 (88.24)	0.000
No	3750 (26.81)	10,236 (73.19)	
Received Antenatal Care (*N* = 7753)			
Yes	513 (10.16)	4538 (89.84)	0.000
No	422 (15.62)	2280 (84.38)	
Received TT Injection (*N* = 7753)			
Yes	491 (10.72)	4090 (89.28)	0.000
No	444 (14.00)	2728 (86.00)	
Place of Residence (*N* = 44,207)			
Urban	968 (13.02)	6466 (86.98)	0.000
Rural	6336 (17.23)	30,437 (82.77)	
Wealth Index(*N* = 44,207)			
Poorest	2193 (20.75)	8375 (79.25)	0.000
Second	1743 (18.61)	7625 (81.39)	
Middle	1479 (16.68)	7390 (83.32)	
Fourth	1184 (14.08)	7227 (85.92)	
Richest	705 (10.08)	6286 (89.92)	

Logistic regression was used to determine the effect of antenatal care and social well-being on ENM. According to the findings of logistic regression model, women who received antenatal care are likely to have 18.0% lower odds of ENM (OR = 0.82, CI = (0.71–0.95)) compared to women who did not and this difference is statistically significant. The odds of experiencing ENM for the respondents who received TT injection during pregnancy is significantly 21.0% lower (OR = 0.79, CI = (0.69–0.91)) compared to unvaccinated respondents. This study found significant impact of school attendance of the women on ENM compared with women who ever attended school having 54.0% lower odds of ENM (OR = 0.46, CI = (0.39–0.53)) compared to women who never attended school. In terms of place of residence, the urban respondents reported that they experienced 20.0% lower odds of ENM (OR = 0.80, CI = (0.64–0.99) compared to rural women. In terms of wealth quintile, woman from the richest families are at 45.0% lower odds of experiencing ENM (OR = 0.55, CI = (0.42–0.72) compared to the poorest, with the difference being statistically significant (Table 3).

**Table 3:** Odds ratios of selected explanatory variables to predict Childhood mortality/Early stage neo-natal mortality

Predictor variables	Childhood mortality/early stage neo-natal mortality	
	Odds ratio	CI (*p* value)
Received Antenatal Care		
Yes	0.82	0.71–0.95 (0.009)
No	1.00	
Received TT Injection		
Yes	0.79	0.69–0.91 (0.001)
No	1.00	
Ever Attended School		
Yes	0.46	0.39–0.53 (0.000)
No	1.00	
Place of Residence		
Urban	0.80	0.64–0.99 (0.050)
Rural	1.00	
Wealth Index		
Richest	0.55	
Poorest	1.00	0.42–0.72 (0.000)
Constant	0.34	
Model chi-square	205.72	
*p*-value	0.000	
N	7753	

## Discussion

Along with other socioeconomic factors, the antenatal period is significantly important for the health of mother and her future offspring. Child survival can be improved by improving care during antenatal period. The present study examined the effects of antenatal care and social well-being on early neonatal mortality (ENM) in Bangladesh. In this study the Bangladesh Multiple Indicator Cluster Survey data was used which was conducted from December 2012 to March 2013 with the collaboration of the Bangladesh Bureau of Statistics (BBS) and Ministry of Planning. The survey used the multistage stratified sampling method for collecting data. This is one of the few studies that focus on the impact of antenatal care and social well-being on early neonatal mortality using a large scale secondary data set.

The results of this study give an overall idea about how proper antenatal care and social well-being can reduce the early neonatal mortality in Bangladesh. The study population of this research was 15–49 years aged group women and among these 51,627 women 44,207 were ever married and had ever given birth. Among these ever married women, 17.0% had history of ENM in their entire reproductive age. To find out the causes of death this study examined several possible factors that could contribute majorly.

The multivariate analysis has been applied to determine the effect of antenatal care and social well-being on early neonatal mortality. Findings from logistic regression model showed that women who received antenatal care, compared to those who did not, experienced 18.0% lower odds of early neonatal mortality; similar findings have been reported by other researchers [11–17]. In terms of wealth quintile, women from the richest family are likely to have 45.0% lower odds of ENM than their poorest counterparts. This finding is consistent with that of other researchers [12, 17–19].

Receiving proper vaccination is an important factor to reduce early neonatal mortality. Findings of this study shows that odds of experiencing ENM for the respondents who received TT injection during pregnancy is significantly 21.0% lower compared to unvaccinated respondents. This finding related to immunization is consistent with that of other researcher [4]. Similar to other socio-demographic factors, place of residence also creates an effect in experiencing early neonatal mortality. In terms of place of residence, based on the findings of this study, the urban respondents reported that they experienced 20.0% lower odds of ENM compared to rural women; similar findings have been reported by other researchers [1, 20, 21].

School attendance is also a significant factor affecting the respondents’ risk of experiencing early neonatal mortality. This study found significant impact of school attendance of the women on ENM with women who ever attended school having 54.0% lower odds of ENM compared to women who never attended school; this finding related to educational attainment is consistent with that of other researchers [1, 14, 22]. So, education is an important factor to reduce early neonatal mortality because having proper education will encourage all pregnant mothers to engage in effective health care seeking behavior that will ultimately reduce neonatal deaths.

## Conclusion

Findings of this paper suggest that women’s antenatal care, social well-being and TT vaccination have a significant role in lowering ENM. This study recommends that for reducing early neonatal mortality (ENM) we should ensure proper antenatal care during pregnancy. Another issue is that the people who are richest and educated will be able to prevent early neonatal mortality by taking the necessary proper actions. So, socio-economic development is also important to address this issue properly. If all the measures taken sufficiently and with alacrity, it will be possible lower ENM which will impact child mortality favorably.

### Limitations

There are several limitations of the present study, i.e. the analysis of the study had to depend on the variables available in the MICS 2012–2013. The issue under study is very complicated and difficult to measure. For that, we had to use indirect and proxy indicators to complete the analysis. However, we were careful in constructing proxy indicators, conducting analysis and interpretation of the data.

## Abbreviations

BBS: Bangladesh Bureau of Statistics
CI: Confidence interval
ENM: Early neonatal mortality
MDGs: Millennium development goals
MICS: Bangladesh multiple indicator cluster survey
OR: Odds ratio
SDGs: Sustainable development goals
WHO: World Health Organization

## References

[1] Mustafa HE, Odimegwu C (2008). Socioeconomic determinants of infant mortality in Kenya: analysis of Kenya DHS 2003. J Humanit Soc Sci.

[2] Gage TB (2013). Maternal education, birth weight, and infant mortality in the United States. Demography.

[3] Aquino R (2009). Impact of the family health program on infant mortality in Brazilian municipalities. Am J Public Health.

[4] Sayem AM (2011). Achieving the millennium development goal for under-five mortality in Bangladesh: current status and lessons for issues and challenges for further improvements. J Health Popul Nutr.

[5] GoB B, SID, UNICEF (2015). Multiple Indicator Cluster Survey, 2012–2013.

[6] Health Bulletin (2017). Government of the People's Republic of Bangladesh, Ministry of Health and Family Welfare, MIS.

[7] UNICEF. Neonatal Mortality. Online (Access on 12 July 2017). https://data.unicef.org/topic/child-survival/neonatal-mortality/.

[8] Ahmad OB (2000). The decline in child mortality: a reappraisal. Bull World Health Organ.

[9] Kim D, Saada A (2013). The social determinants of infant mortality and birth outcomes in Western developed nations: a cross-country systematic review. Int J Environ Res Public Health.

[10] UNICEF (2014). World bank; United Nations. Levels & trends in child mortality report 2014: estimates developed by the UN InterAgency Group for Child Mortality Estimation.

[11] Van Eijk AM (2006). Use of antenatal services and delivery care among women in rural western Kenya: a community based survey. Reprod Health.

[12] Hollowell J (2011). The effectiveness of antenatal care programmes to reduce infant mortality and preterm birth in socially disadvantaged and vulnerable women in high-income countries: a systematic review. BMC Pregnancy Childbirth.

[13] Zafari M (2012). A study the factors affecting under the age of 5 years child mortality. Int J Prev Treat.

[14] Owais A (2013). Maternal and antenatal risk factors for stillbirths and neonatal mortality in rural Bangladesh: a case-control study. PloS One.

[15] Singh A, et al. Do antenatal care interventions improve neonatal survival in India? Health Policy Plan. 2013:czt066. 10.1093/heapol/czt066 PMC4471439 24038077

[16] Trujillo JC, et al. Relationship between professional antenatal care and facility delivery: an assessment of Colombia. Health Policy Plan. 2013:czt033. 10.1093/heapol/czt033 23735737

[17] Shera HMMJ, Dar IS (2014). Addressing Corner Solution Effect for Child Mortality Status Measure: An Application of Tobit Model. Int J Acad Res Bus Soc Sci.

[18] Kembo J, Van Ginneken JK (2009). Determinants of infant and child mortality in Zimbabwe: Results of multivariate hazard analysis. Demog Res.

[19] Belizán JM (2012). Neonatal death in low-to middle-income countries: a global network study. Am J Perinatol.

[20] Fottrell E, Osrin D, Alcock G, Azad K, Bapat U, Beard J, et al. Cause-specific neonatal mortality: analysis of 3772 neonatal deaths in Nepal, Bangladesh, Malawi and India. Arch Dis Child Fetal Neonatal Ed. 2015, 2014. 10.1136/archdischild-2014-307636 PMC4552925 25972443

[21] Nahar J, Zabeen B, Akhter S, Azad K, Nahar N (2007). Neonatal morbidity and mortality pattern in the special care baby unit of BIRDEM. Ibrahim Med Coll J.

[22] Khatun F, Rasheed S, Moran AC, Alam AM, Shomik MS, Sultana M (2012). Causes of neonatal and maternal deaths in Dhaka slums: implications for service delivery. BMC Public Health.

